# Role of Baseline and Post-Therapy 18F-FDG PET in the Prognostic Stratification of Metastatic Castration-Resistant Prostate Cancer (mCRPC) Patients Treated with Radium-223

**DOI:** 10.3390/cancers12010031

**Published:** 2019-12-20

**Authors:** Matteo Bauckneht, Selene Capitanio, Maria Isabella Donegani, Elisa Zanardi, Alberto Miceli, Roberto Murialdo, Stefano Raffa, Laura Tomasello, Martina Vitti, Alessia Cavo, Fabio Catalano, Manlio Mencoboni, Marcello Ceppi, Cecilia Marini, Giuseppe Fornarini, Francesco Boccardo, Gianmario Sambuceti, Silvia Morbelli

**Affiliations:** 1 Nuclear Medicine Unit, IRCCS Ospedale Policlinico San Martino, 16132 Genoa, Italy; selene.capitanio@hsanmartino.it (S.C.); sambuceti@unige.it (G.S.); silviadaniela.morbelli@hsanmartino.it (S.M.); 2 Department of Health Sciences (DISSAL), University of Genova, 16132 Genoa, Italy; isabella.donegani@gmail.com (M.I.D.); albertomiceli23@gmail.com (A.M.); stefanoraffa@live.com (S.R.); vitti.martina@hotmail.it (M.V.); cecilia.marini@unige.it (C.M.); 3 Academic Unit of Medical Oncology, IRCCS Ospedale Policlinico San Martino, 16132 Genoa, Italy; elisa.zanardi@unige.it (E.Z.); laura.tomasello@hsanmartino.it (L.T.); fboccardo@unige.it (F.B.); 4 Department of Internal Medicine and Medical Specialties (DIMI), University of Genoa, 16132 Genoa, Italy; 5 Internal Medicine Unit, IRCCS Ospedale Policlinico San Martino, 16132 Genoa, Italy; roberto.murialdo@hsanmartino.it; 6 Oncology Unit, Villa Scassi Hospital, 16149, Genova, Italy; alessia.cavo@asl3.liguria.it (A.C.); manlio.mencoboni@asl3.liguria.it (M.M.); 7 Medical Oncology Unit, IRCCS Ospedale Policlinico San Martino, 16132 Genoa, Italy; catalan.fab@gmail.com (F.C.); giuseppe.fornarini@hsanmartino.it (G.F.); 8 Clinical Epidemiology Unit, IRCCS Ospedale Policlinico San Martino, 16132 Genoa, Italy; marcello.ceppi@hsanmartino.it; 9 CNR Institute of Molecular Bioimaging and Physiology (IBFM), 20090 Segrate (MI), Italy

**Keywords:** Radium-223, positron emission tomography, FDG, castrate resistant prostate cancer

## Abstract

Radium-223 dichloride (Ra223) represents the unique bone-directed treatment option that shows an improvement in overall survival (OS) in metastatic castrate resistant prostate cancer (mCRPC). However, there is an urgent need for the identification of reliable biomarkers to non-invasively determine its efficacy (possibly improving patients’ selection or identifying responders’ after therapy completion). 18F-Fluorodeoxyglucose (FDG)-avidity is low in naïve prostate cancer, but it is enhanced in advanced and chemotherapy-refractory mCRPC, providing prognostic insights. Moreover, this tool showed high potential for the evaluation of response in cancer patients with bone involvement. For these reasons, FDG Positron Emission Tomography (FDG-PET) might represent an effective tool that is able to provide prognostic stratification (improving patients selection) at baseline and assessing the treatment response to Ra223. We conducted a retrospective analysis of 28 mCRPC patients that were treated with Ra223 and submitted to bone scan and FDG-PET/CT for prognostic purposes at baseline and within two months after therapy completion. The following parameters were measured: number of bone lesions at bone scan, SUVmax of the hottest bone lesion, metabolic tumor volume (MTV), and total lesion glycolysis (TLG). In patients who underwent post-therapy 18F-Fluorodeoxyglucose Positron Emission Tomography/Computed Tomography (FDG-PET/CT), (20/28), PET Response Criteria in Solid Tumors (PERCIST), and European Organization for Research and Treatment of Cancer (EORTC) criteria were applied to evaluate the metabolic treatment response. The difference between end of therapy and baseline values was also calculated for Metabolic Tumor Volume (MTV), TLG, prostate-specific antigen (PSA), alkaline phosphatase (AP), and lactate dehydrogenase (LDH) (termed deltaMTV, deltaTLG, deltaPSA, deltaAP and deltaLDH, respectively). Predictive power of baseline and post-therapy PET- and biochemical-derived parameters on OS were assessed by Kaplan–Meier, univariate and multivariate analyses. At baseline, PSA, LDH, and MTV significantly predicted OS. However, MTV (but not PSA nor LDH) was able to identify a subgroup of patients with worse prognosis, even after adjusting for the number of lesions at bone scan (which, in turn, was not an independent predictor of OS). After therapy, PERCIST criteria were able to capture the response to Ra223 by demonstrating longer OS in patients with partial metabolic response. Moreover, the biochemical parameters were outperformed by PERCIST in the post-treatment setting, as their variation after therapy was not informative on long term OS. The present study supports the role of FDG-PET as a tool for patient’s selection and response assessment in mCRPC patients undergoing Ra223 administration.

## 1. Introduction

Radium-223 dichloride (Ra223) is a radionuclide that selectively binds to areas of increased bone turnover in bone metastases. The short range of energy delivery that is typical of its apha-emission makes Ra223 an effective therapeutic approach for symptomatic patients with bone metastatic castration-resistant prostate cancer (mCRPC) [1].

Besides documenting its effect on pain palliation, the pivotal phase 3 Alpharadin in Symptomatic Prostate Cancer Patients (ALSYMPCA) trial demonstrated the prognostic benefit that is induced by this radio-metabolic approach that prolonged overall survival (OS) and delayed time to the first symptomatic skeletal event with respect to the placebo arm [1]. However, in the last few years, some retrospective studies suggested that the OS benefit in real-life patients might be lower than that reported in the ALSYMPCA study [2,3,4,5]. These evidences might be explained either by the suboptimal sequence of Ra223 use in the castrate-resistant phase or as the result of a suboptimal selection of patients with poorer prognostic clinical characteristics. A similar consideration also applies to the monitoring of therapy effectiveness: a number of limitations hamper the direct evaluation of bone lesion evolution asking for a combined approach while using clinical, biochemical (e.g. prostate-specific antigen (PSA), serum alkaline phosphatase (AP)), and imaging assessments [6]. However, this approach is also hampered by the so-called flare effect: the early and transient rise in the PSA levels or tracer uptake at bone scan, followed by a later decline that is commonly observed in prostate cancer patients treated with chemotherapy, luteinizing hormone-releasing hormone, and even Ra223 [7]. Indeed, the role of imaging for capturing response to Ra223 is still unclear and data regarding the most accurate modality for evaluating treated patients are lacking.

In this framework, while 18F-Fluorodeoxyglucose Positron Emission Tomography/Computed Tomography (FDG-PET/CT) is not indicated in naïve prostate cancer, as they have low FDG-avidity, mCRPC patients, and, even more, chemotherapy-refractory mCRPC are characterized by higher FDG uptake. For this reason, FDG has been proposed as a tool that is able to aggressively identify disease in mCRPC [8,9,10,11], and more in general molecular imaging with PET has high potential for the evaluation of response in cancer patients with bone involvement [12]. Based on these considerations, FDG-PET/CT might represent an effective tool for measuring disease burden and aggressiveness at baseline and for assessing the treatment response to Ra223 in mCRPC.

In the present retrospective, single center study, we aimed to evaluate the potential role of FDG-PET/CT in patient’s selection and evaluation of response in a cohort of mCRPC treated with Ra223. To this aim, FDG-avid metabolic tumor burden at baseline and post-therapy of patients that were treated with Ra223 was computed and its relationship with biochemical parameters as well as progression-free (PFS) and overall survival (OS) was assessed.

## 2. Patients and Methods

### 2.1. Study Protocol

We conducted a retrospective review of mCRPC patients treated with Ra223 and submitted to FDG-PET/CT at baseline and within two months after therapy between September 2016 and November 2018 in our institution. All of the patients with an available baseline FDG-PET/CT scan were included (regardless of the availability of a post-therapy scan). By contrast, patients with an available post-therapy were only included when a baseline scan was available in the 30 days preceding Ra223 administration. Patients were candidate to FDG-PET/CT, in agreement with the evidence reported in national guidelines of the Italian Association of Medical Oncology (AOIM) on the emerging role of FDG-PET/CT in patients with mCRPC or in patients with poorly differentiated prostate cancer (Gleason score ≥ 8) [13,14]. According to our standard procedure, all of the patients signed a written informed consent form, which encompassed the use of anonymized data for retrospective research purposes, before each PET/CT scan.

All of the patients obviously had to fulfill all of the inclusion criteria for Ra223 treatment and they were treated with standard Ra223 regimen encompassing six administrations every four weeks (55 KBq/kg, intravenously). According to the established selection criteria for Ra223 patient’s selection [13], in the previous four weeks preceding the first administration, each patient underwent contrast enhanced CT (ceCT) and bone scan, in order to select mCRPC patients with symptomatic bone metastases in the absence of visceral metastases (with the exception for lymph nodes with maximum diameter < 3 cm). Similarly, a bone marrow reserve fulfilling the hematologic criteria necessary for administering Ra223 and Eastern Cooperative Oncology Group (ECOG) performance status of 0–1 (life expectancy greater than six months) were verified [13]. Clinical and biochemical variables at baseline, in course and at the end of therapy, also had to be available at the time of our study. These variables included: ECOG performance status, PSA, AP, and lactate dehydrogenase (LDH). During treatment, the patients were maintained under androgen deprivation therapy.

### 2.2. Imaging Procedures

Bone scan and FDG-PET/CT were performed according to European Association of Nuclear Medicine (EANM) Guidelines [15,16], as detailed in the Supplementary Materials. Briefly, bone scan was acquired 3 h after the injection of 740 MBq of 99mTc-methilen diphosphonate. A dual-head gamma camera (Millennium; GE Healthcare, Boston, USA) that was equipped with a parallel-hole, low-energy, high-resolution collimator on a 10% energy window centered on the 99mTc photopeak was used to acquire planar whole-body acquisitions, and, if needed, Single Photon Emission Computed Tomography (SPECT) images. FDG-PET/CT was performed while using a 16-slices PET/CT hybrid system (Hirez-Biograph 16, Siemens Medical Solutions, Munich, Germany). After blood glucose levels measurement (to ensure blood glucose levels <160 mg/dL), fasting patients underwent the intravenous injection of 300–400 MBq of FDG. The patients were asked to drink 500 mL of water 1h prior to image acquisition and empty the bladder just before the acquisition start. Imaging started 60 ± 15 minutes after tracer administration. The acquired PET data were reconstructed into a 128 × 128 matrix using an iterative reconstruction algorithm (three iterative steps, eight subsets). Raw images were scatter-corrected and processed while using a three-dimensional (3D) Gaussian filter, while CT was used for attenuation correction. Image quality control documented a spatial resolution of 4.0 mm full width at half-maximum.

### 2.3. Image Analysis

The bone scan images were qualitatively evaluated, and number of lesions present at baseline and after therapy was recorded. The following semiquantitative parameters were computed on both baseline and post-therapy FDG-PET/CT scans: maximum standard uptake value (SUVmax) of the hottest bone lesion, Metabolic Tumor Volume (MTV) and Total Lesion Glycolysis (TLG). MTV was estimated from the volumes of interest SUV isocontours that were automatically drawn by setting a relative threshold of 40% of the SUVmax [17]. TLG was calculated as SUVmean × MTV. All of the semiquantitative variables were estimated at baseline and after therapy to calculate their difference (termed deltaMTV and deltaTLG, respectively). However, deltaSUVmax was not calculated, as the hottest bone lesion at post-therapy FDG-PET/CT might not correspond to the hottest lesion at baseline. The metabolic response to Ra223 was also evaluated by two independent readers (MB, SM) while using PET Response Criteria in Solid Tumors (PERCIST) and European Organization for Research and Treatment of Cancer (EORTC) criteria [18,19], see also Supplementary Materials for details on PERCIST and EORTC criteria). The PET readers were blinded to ceCT and bone scan results. DeltaPSA, deltaAP, and deltaLDH were also calculated as the difference between post-therapy and baseline levels.

### 2.4. Statistical Analysis

PFS and OS curves were computed according to Kaplan–Meier with the Log Rank test determining statistical significance. This analysis was performed by dividing the population into tertiles for all tested variables, with the only exception of bone scan lesions whose number was divided in ≤ 6, 6–20, and > 20, as performed in a sub-analysis of the ALSYMPCA study [1]. A binary classification of response to therapy was applied to PERCIST scores due to the small numbers involved, as previously described [20]. In particular, patients showing partial metabolic response (PMR) were considered as “responders”, while stable metabolic disease (SMD) and progressive metabolic disease (PMD) were classified as “non-responders”. A set of univariate and multivariate Cox proportional hazard models were fitted to the data to assess the predictive value of baseline parameters: in univariate analyses, OS and PFS were modeled as a function of each tested variable. Subsequently, all of the tested variables were tentatively included in a multivariate Cox’s model by means of a step-down (backward) procedure, based on the likelihood ratio test: variables with a *p* value < 0.1 were removed from the model. Proportionality assumptions were assessed, as previously described [21]. Analyses were conducted with IBM-SPSS release 23 (IBM, Armonk, USA).

## 3. Results

The interrogation of our database identified 28 mCRPC patients who met the inclusion criteria and they were submitted to baseline FDG-PET/CT. Within this cohort, six patients died before treatment completion, while two refused the post-therapy FDG-PET/CT, but were still alive at the time of database interrogation. Accordingly, functional imaging at both time points was available in 20 subjects. The median interval between the two PET/CT scan was 213 days. Table 1 summarizes the overall patient’s characteristics. Once completed the treatment protocol, the 20 remaining patients were monitored for twelve months after the post-therapy PET/CT. During this one-year follow-up, further 10/20 (50%) patients died. Figure S1 reports OS in the whole study cohort (since treatment start).

### 3.1. Prognostic Role of Baseline Parameters

**Figure 1:**
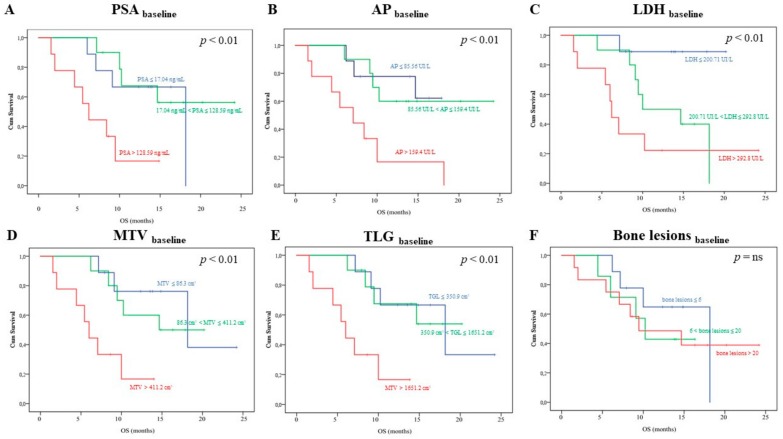
Kaplan–Meyer analysis including baseline biochemical and imaging variables. Each panel show the overall survival (OS) curves depending on baseline prostate-specific antigen (PSA), alkaline phosphatase (AP), lactate dehydrogenase (LDH), metabolic tumor volume (MTV), total lesion glycolysis (TLG), and bone lesions, respectively. In each case, but Panel F, patients were divided in three tertiles (first tertile: blue; second tertile: green; third tertile: orange). Following a subgroup analysis proposed in the ALSYMPCA study, in Panel F, patients were divided in three subgroups according to the number bone lesions at bone scan in < 6 (blue), 6–19 (green), and > 20 (orange) bone metastases, respectively.

As shown in Figure 1, Kaplan–Meyer analysis revealed that the biochemical parameters PSA, AP, and LDH, as well as the FDG imaging ones MTV and TLG provided a risk stratification power. In fact, the death rate was highest in mCRPC patients’ candidates to Ra223 clustered within the highest tertile for each of them (*p* < 0.01, respectively). By contrast, the whole body bone scan was devoid of this prognostic penetrance, since mortality rate was remarkably similar among patients with low, intermediate, or high number of detectable bone lesions [1]. Univariate analysis that reported a significant association between overall survival and PSA, LDH, and MTV confirmed this observation (*p* < 0.01 each, respectively), as shown in Table 2 (see also Table S1 for the univariate analysis including the same variables as categorial). Multivariate Cox Regression analysis reported that each of these variables was characterized an additive predictive capability (Table 2, see also Table S1 for the multivariate analysis, including the same variables as categorial). By contrast, no baseline clinical or imaging variable was able to predict the PFS.

**Figure 2:**
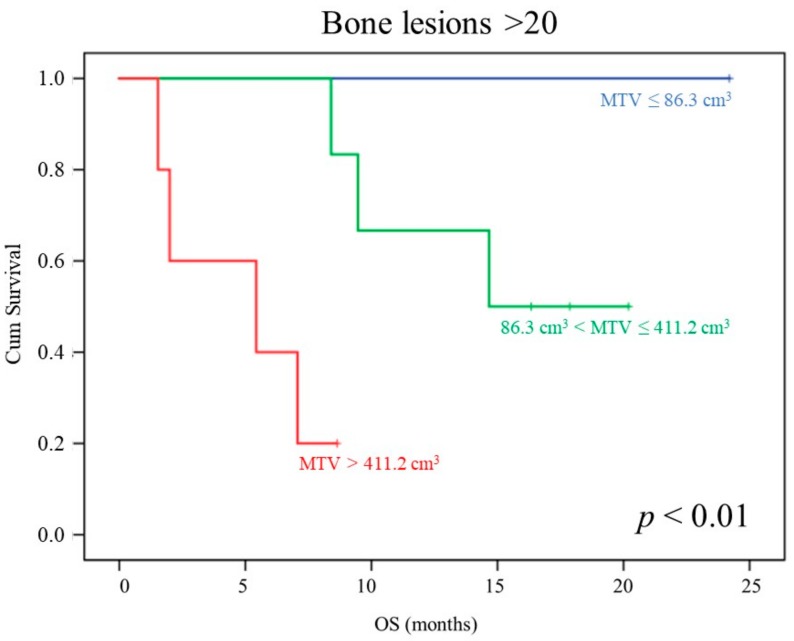
MTV prognostic power in patients showing >20 lesions at bone scan. OS curves depending on baseline MTV (first tertile: blue; second tertile: green; third tertile: orange) in the subgroup of patients showing > 20 bone lesions at bone scan.

Of note, focusing the survival analysis on the 12 patients showing >20 bone lesions at bone scan, neither PSA, AP, nor LDH serum levels significantly predicted OS, while MTV was able to stratify three different prognostic classes (*p* < 0.01, Figure 2). Again, the multivariate analysis confirmed these data (Cox Regression, backward stepwise, LR; *p* < 0.0001).

### 3.2. FDG PET/CT in the Response Evaluation Following Ra223

Table 1 sumamrizes the overall clinical characteristics of the 20 patients who underwent FDG-PET/CT post therapy evaluation. Ra223 administration caused a measurable response in most of the tested variables. In particular, hematopoietic response was confirmed by a decrease in hemoglobin levels and in white blood cells count, while the decrease in platelet number did not reach the statistical significance. AP showed a significant decrease. Nevertheless, the suggested reduction in osteoblastic activity was devoid of any significant risk prediction power, as reported in Supplementary Materials Figure S2, Panel B. The tumor burden indexes showed an opposite response that roughly approached 25% for LDH and almost 300% for PSA. Again, this response was not able to stratify patient’s outcome (Figure S2, Panels A and C), which confirmed a relevant interference of the so-called “flair effect” after radiometabolic treatment of bone lesions.

**Figure 3:**
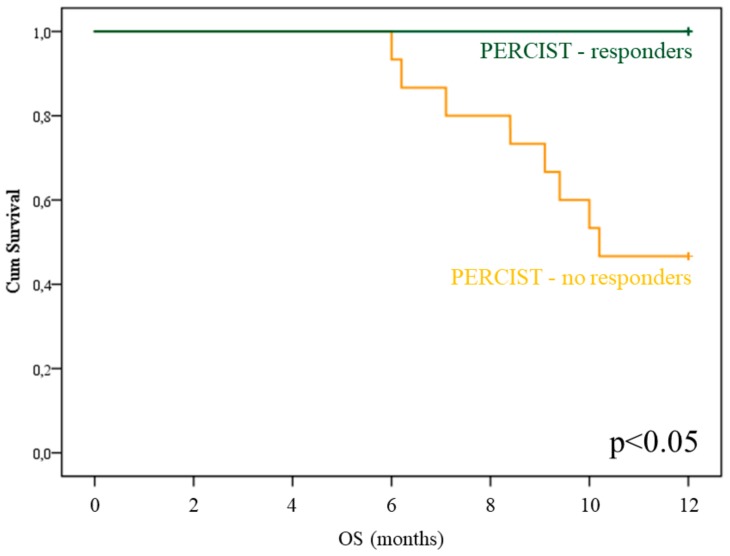
PET Response Criteria in Solid Tumors (PERCIST) prognostic power in Radium-223 treated patients. OS curves depending on PERCIST score (PMR: blue; SMD+PMD: green) in the subgroup of patients submitted to post-therapy 18F-Fluorodeoxyglucose Positron Emission Tomography/Computed Tomography (FDG-PET/CT).

A completely different consideration applied to imaging indexes of treatment effectiveness. PERCIST scores significantly predicted OS (Figure 3, *p* < 0.05), while this prognostic penetrance did not reach statistical significance for EORTC, deltaMTV, and deltaTLG evaluations.

**Figure 4:**
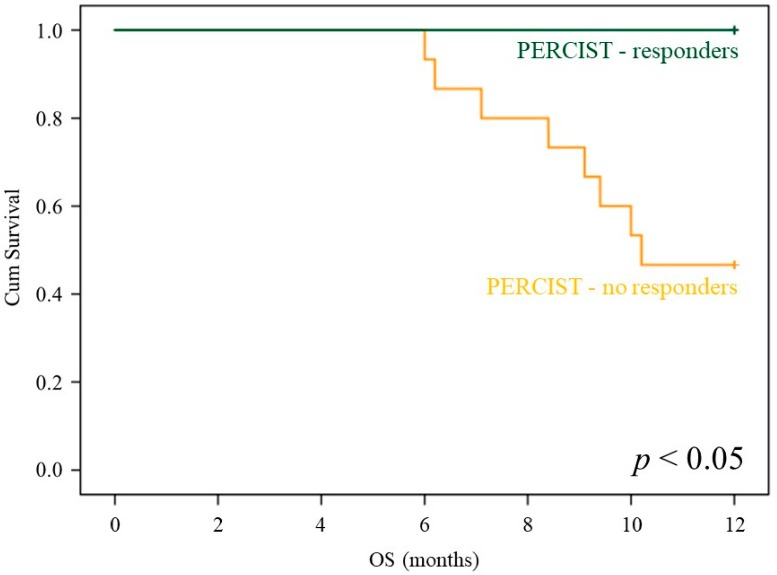
PERCIST prognostic stratification in deltaPSA classes. OS curves depending on PERCIST score (PMR: blue; SMD+PMD: green) in the subgroups of patients submitted to post-therapy FDG-PET/CT classified on the basis of the deltaPSA class.

**Figure 5:**
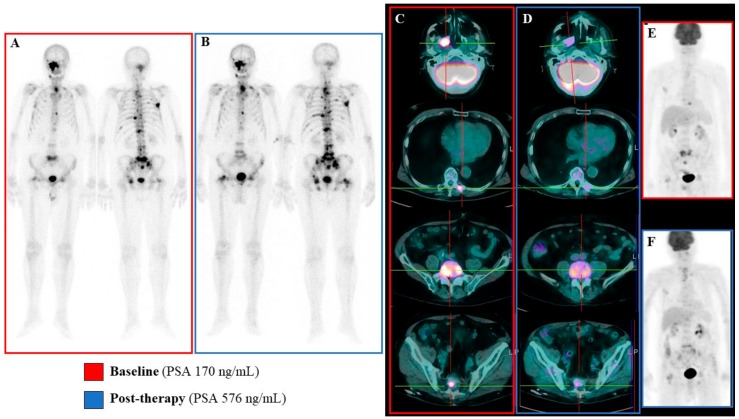
Emblematic example of mismatch between metabolic and biochemical/bone scan response. In this emblematic case a mismatch between metabolic data and biochemical/bone scan results was observed. At baseline the bone scan revealed the presence of several osteoblastic bone lesions (Panel **A**) that were mainly FDG-avid (Panels **C** and **E**). The post-therapy evaluation, performed six weeks after Ra223 protocol completion, showed the persistence of osteoblastic bone lesions that were occasionally more extended and diphosphonate-avid (Panel **B**). This finding was paralleled by increased serum PSA levels (576 vs 170 ng/mL at baseline). However, the post-therapy FDG PET/CT showed a significant reduction in bone lesions FDG uptake, leading to a Partial Metabolic Response according to PERCIST criteria (Panels **D** and **F**).

Multivariate Cox analysis, including deltaPSA, deltaAP, deltaLDH, and PERCIST score, confirmed the additive value of PERCIST score with respect to all of the remaining variables (*p* = 0.012). In agreement with this finding, the imaging index virtually preserved its capability to predict the response to treatment in patients with the highest increase in PSA after Ra223 administration, as suggested by the almost significant p value (*p* = 0.1 in each subgroup, see also Figure 4) and the emblematic example reported in Figure 5.

## 4. Discussion

The present results support the role of both baseline and post-therapy FDG-PET/CT in mCRPC patients treated with Ra223. In fact, MTV evaluation of baseline metabolically active skeletal disease burden was able to predict OS with an additive independent value, as documented by multivariate Cox regression analysis. By contrast, the number of lesions, as assessed by bone scan, was characterized by a largely less evident prognostic penetrance. Similarly, biochemical biomarkers PSA, AP, and LDH confirmed their predictive value when assessed before Ra223 treatment. However, their response to therapy was virtually devoid of any prognostic penetrance. By contrast, PET-based evaluations and, in particular, PERCIST criteria, were able to predict the response to Ra223 by demonstrating a lower mortality rate in patients with partial metabolic response.

### 4.1. Patient’s Selection for Ra223

Patients’ selection is a crucial point when discussing the role of Ra223 in mCRPC patients. In 2018, the European Medicines Agency (EMA) issued a specific note on the indications for treatment with Ra223 in a review of an ongoing phase 3 trial by the EMA’s Pharmacovigilance Risk Assessment Committee, basically moving Ra223 to a third-line of treatment of mCRPC patients (EMA/500948/2018; https://www.ema.europa.eu/en/documents/press-release/ema-restricts-use-prostate-cancer-medicine-xofigo_en.pdf). The observation of increased the risk of fractures, in the absence of statistically significant survival benefit in patients with <6 bone lesions enrolled in the ERA-223 study, receiving Ra223 in combination with abiraterone acetate plus prednisone/prednisolone, motivated this action [22]. In fact, patients with lower skeletal disease burden might have a larger proportion of Ra223 homing to healthy bone, as compared to patients with extensive bone metastases. Indeed, sites of bony metastases are known to have a higher bone turnover and the sequestration of administered Ra223 (the so-called “sink-effect”), which might limit Ra223 deposition in the non-diseased skeleton [23,24]. However, besides the mere number of bone lesions, a huge variability in Ra223-avid bone disease can be observed. Preclinical prostate cancer models showed that the short range of alpha particles results in a lower tumor control-rate of large metastatic deposits as compared to near eradication of small lesions [25]. These data suggest the inadequacy of the mere bone lesion number count in the identification of the right candidate to Ra223.

Previous studies showed that the quantification of skeletal tumor burden by means of bone scintigraphy or 18F-NaF PET/CT might predict patients’ OS and reflect the risk of hematological toxicity related to Ra223 administration [26,27]. The present study is in keeping with these evidences as also FDG-PET/CT-derived tumor volume and not the mere number of bone lesions predicted patients’ prognosis. However, the present findings further extend this evidence by demonstrating the specific weight of metabolic active tumor burden.

Indeed, the higher predictive value of 18F-FDG PET/CT with respect to bone scan might be due to its capability to capture bone marrow involvement, which is obviously underestimated by the simple osteoblastic bone disease detected by bone scan (and even by 18F-NaF-PET/CT) [28]. On the other hand, the detection of FDG-avid de-differentiated and non-osteoblastic bone disease [10,29,30] might mirror greater tumour aggressiveness, also possibly predicting the lower Ra223 accumulation and the consequent low-response rate. Moreover, as the added prognostic value of the extent of metabolic active disease was preserved in patients showing >20 bone lesions at bone scan, FDG-PET/CT might play a role in the selection of patients with larger tumour burden. This finding is of potential clinical relevance, since high-volume bone disease predicts higher degrees of haematological toxicity and the selection of this group of patients for Ra223 should be even more thoughtful [26,31]. Finally, if confirmed in larger studies, the highlighted FDG-PET/CT capability rule out high-risk candidates to Ra223 might be relevant for the cost/effectiveness of Ra223 administration, thus reducing the risk of ineffective treatments in patients with worst prognosis and higher risk of hematological toxicity.

### 4.2. Post-Therapy Assessment

In the ALSYMPCA trial, Ra223 showed a modest effect on serum PSA levels, which reached a ≥30% reduction in just 16% of treated patients, despite improving OS [1]. Moreover, a minor correlation between OS and PSA changes at week 12 was observed [32] and, especially during the first three months of treatment, possible flare phenomena have been described [33,34,35]. AP and LDH kinetics under Ra223 administration has been proposed as an alternative approach to the PSA monitoring [1,36]. However, post-hoc analyses from the ALSYMPCA trial showed that these biomarkers did not meet the needed statistical surrogacy requirement for OS [32]. Therefore, the low predictive value of these biochemical biomarkers raises the urgent need for the identification of novel biomarkers, even if the dynamic monitoring of AP and LDH serum levels is still recommended during Ra223 administration.

Even though imaging was not routinely performed in the ALSYMPCA trial, imaging with bone-targeted radiopharmaceuticals has been proposed as a tool for monitoring treatment response when clinically indicated [1]. However, bon flare phenomenon resulting in false-positive tracer uptake, even in responsive cases, has been described [23,37,38], which complicates the evaluation of response at a single patient level. Recently, the 2 + 2 rule has been proposed for overcoming this limitation of osteotropic radiotracers [26,34,39]. However, its reliability in the clinical setting still needs to be validated. On the other hand, ceCT scan can be used in case of suspicious disease progression. However, in prostate cancer metastatic lymph nodes can be normal sized resulting false-negative at anatomic imaging [40,41], profoundly underpowering the accuracy of this tool in the evaluation of response to Ra223. In this scenario, 18F-Fluorocholine or 68Ga-PSMA PET/CT have been recently proposed as promising tools for monitoring the response to Ra223 treatment [31,42,43,44,45].

To the best of our knowledge, a case report of mCRPC [46], a small case series [47], and a phase IIa metastatic breast cancer study represent the sole available reports on FDG-PET/CT used for the evaluation of Ra223 response [48]. In the present study, the application of PERCIST criteria to FDG-PET/CT images overpowered bone scan and biochemical parameters in the evaluation of response to Ra223 treatment. Moreover, a trend in the post-treatment prognostic assessment was maintained when the PERCIST classes were analyzed in patients experiencing an increase in PSA levels. This latter finding suggests the potential role for FDG-PET/CT in the identification of the PSA-flare phenomenon. The observed discordance in the bone imaging patterns between bone scan and FDG-PET/CT after Ra223 administration might be related to bone marrow metastases, which cannot be detected by bone scintigraphy. Importantly, these discordances have been observed, not only in case of disease progression, but also in metabolic responders, although the magnitude of this discordance seems to be larger in cases of progression. On one side, this finding fits with the acknowledged limited efficacy of Ra223 in bone marrow metastases, as consistent with its mode of action [34,49,50,51]. On the other hand, it highlights a need for optimal clinical management of Ra223-treated patients who present with marrow metastases at baseline.

### 4.3. Limitations

The present study has some drawbacks. First, the relatively small population. However, its monocentric nature represents one of its strengths. Accordingly, all of the enrolled patients were submitted to FDG-PET/CT while using the same PET/CT scanner avoiding the possible influence of inter-scanner variability on PET results. Second, in the multivariate analyses, we did not consider other potential prognostic factors in the cohort able to influence OS and, consequently, the impact of the different response criteria investigated. Finally, bone scan images were qualitatively evaluated, while the application of semi-quantification tools [26,52] might have provided the estimation of the “osteoblastic tumor volume”, improving the comparison between bone scan and metabolic parameters that were obtained by FDG-PET/CT.

## 5. Conclusions

FDG-PET/CT can represent a widely available method for directly measuring active de-differentiated tumor burden in mCRPC patients’ candidates to Ra223, providing prognostic information overpowering biochemical and other imaging parameters. After treatment, it can assess tumor load reduction particularly in patients showing increased PSA, helping in the differential diagnosis between progression and pseudoprogression (related to the PSA flare phenomenon).

## Figures and Tables

**Table 1:** Patients characteristics.

	Baseline (*n* = 28)	End of Therapy (*n* = 20)	*p*
**Age**	76.71 ± 7.1	-	-
**Gleason score at diagnosis**	-	-	-
5	1 (3.6%)	-	-
6	6 (21.4%)	-	-
7	9 (32.1%)	-	-
8	9 (32.1%)	-	-
9	2 (7.2%)	-	-
10	1 (3.6%)	-	-
**Time evolution of prostate cancer (months)**	90.59 ± 156.3	-	-
**Time gap from diagnosis to mCRPC (months)**	40.22 ± 54.7	-	-
**Number of previous systemic therapies for mCRPC**	-	-	-
1	8 (28.6%)	-	
2	6 (21.4%)	-	
3	7 (25%)	-	
4	6 (21.4%)	-	
5	1 (3.6%)	-	
**ECOG score**	-	-	-
0	5 (17%)	3 (15%)	*p* = ns
1	23 (82%)	17 (85%)	*p* = ns
**Number of Ra223 administered doses**	-	5.3 ± 1.1	-
**Pain response**	-	6 (30%)	-
**Lab tests**	-	-	
Hemoglobin (g/dL)	12.34 ± 1.6	10.87 ± 1.1	*p* < 0.05
White Blood Cells (x10^9^/L)	6.6 ± 2.2	5.5 ± 1.9	*p* < 0.05
Platelets (x10^9^/L)	236 ± 72.9	214.7 ± 84.3	*p* = ns
PSA (ng/mL)	119.25 ± 171.5	347.80 ± 468.1	*p*<0.01
AP (UI/L)	213.3 ± 295.4	133.46 ± 159.1	*p* < 0.05
LDH (UI/L)	265.23 ± 112.1	317.36 ± 241.9	*p* < 0.05
**Bone scintigraphy parameters**	-	-	-
1–6 lesions	7 (25%)	9 (45%)	*p* = ns
6-20 lesions	9 (32%)	3 (15%)	*p* = ns
> 20 lesions	9 (32%)	4 (20%)	*p* = ns
Superscan	3 (10%)	4 (20%)	*p* = ns
**Extraosseus involvement**	-	-	-
Lymph nodes	4 (14.3%)	6 (30%)	
Prostate	2 (7%)	2 (1%)	
**FDG PET/CT paramethers**	-	-	-
SUVmax of the hottest bone lesion	7.25 ± 3.4	8.04 ± 4.3	ns
MTV (cm3)	352.71 ± 384.3	468.01 ± 489.9	*p* < 0.01
TLG	1358.92 ± 1560.9	1877.69 ± 2179.13	*p* < 0.01

**Table 2:** The prognostic role of baseline parameters: uni- and multivariate analyses.

		Univariate Analysis					Multivariate Analysis				
	df	*p*	H.R.	95% CI			*p*	H.R.	95% CI		
**PSA**	1	0.001	1.007	1.003	-	1.010	0.004	1.007	1.002	-	1.011
**AP**	1	0.020	1.001	0.000	-	0.001	0.443	*			
**LDH**	1	0.001	1.009	1.004	-	1.015	0.010	1.009	1.002	-	1.016
**Number of bone lesions**	1	0.960	1.009	−0.002	-	0.019	0.300	*			
**SUVmax**	1	0.976	1.001	−0.055	-	0.057	0.204	*			
**MTV**	1	0.001	1.002	1.001	-	1.003	0.044	1.002	1.000	-	1.003
**TLG**	1	0.134	5 × 10^−005^	0.339	-	0.733	0.411	*			

* = excluded from the final model.

## Supplementary Materials

The following are available online at https://www.mdpi.com/2072-6694/12/1/31/s1: Supplementary Materials, Table S1: prognostic role of baseline parameters as categorical variables: uni- and multivariate analyses. Figure S1: OS in the whole study cohort. Figure S2: Reduction in osteoblastic activity.
