# Supplementary material for: Role of Baseline and Post-Therapy 18F-FDG PET in the Prognostic Stratification of Metastatic Castration-Resistant Prostate Cancer (mCRPC) Patients Treated with Radium-223
Source: Cancers (Basel). 2019 Dec 20;12(1):31. doi: 10.3390/cancers12010031 (PMC7016706; doi:10.3390/cancers12010031)
Supplement: Supplementary file 1 [file cancers-12-00031-s001.pdf]

Supplementary Materials

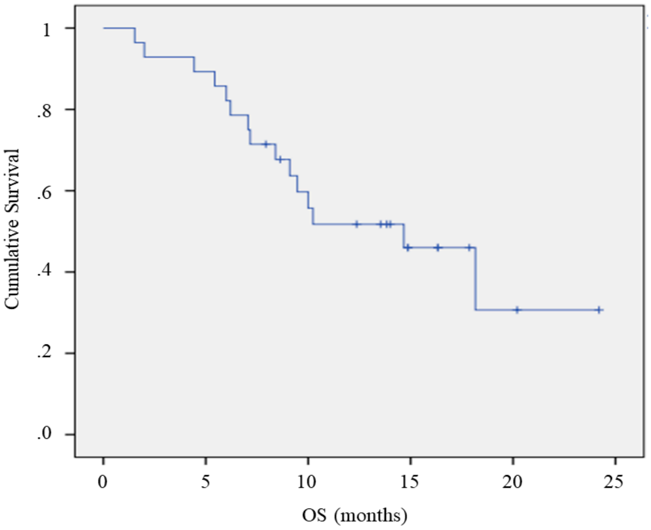

Figure S1. OS in the whole study cohort. OS-overall survival.

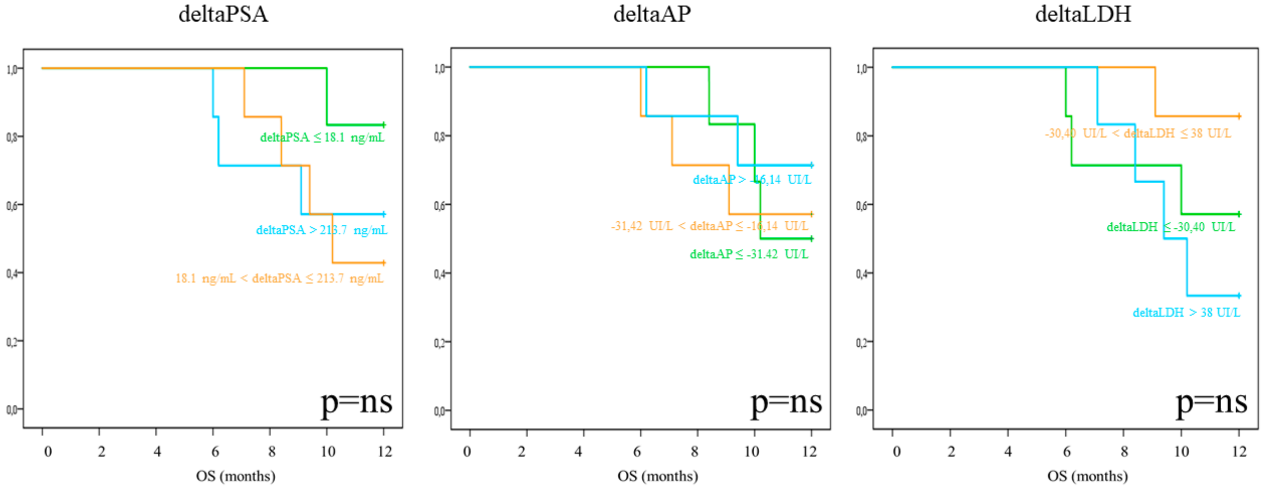

Figure S2. Reduction in osteoblastic activity.

Table S1. Prognostic role of baseline parameters as categorical variables: uni- and multivariate analyses.

	Univariate analysis					Multivariate analysis			
	df	P	H.R.	95% CI		p	H.R.	95% CI	
**PSA$_{cat}$**	1	.041	2.197	1.034	4.668		*		
**AP$_{cat}$**	1	.012	2.600	1.230	5.494		*		
**LDH$_{cat}$**	1	.008	2.740	1.305	5.751	.002	4.468	1.722	11.593
**Bone lesions$_{cat}$**	1	.535	1.207	.666	2.186		*		
**SUVmax$_{cat}$**	1	.383	1.332	.699	2.356		*		
**MTV$_{cat}$**	1	.008	2.886	1.318	6.321		*		
**TLG$_{cat}$**	1	.015	2.540	1.203	5.365	.004	3.651	1.530	8.710

Data presented as continous variables in Table 2 were analysed as categorical variables (cat) in both uni- and multivariate analysis. This analysis was performed by dividing the population into tertiles for all tested variables with the only exception of bone scan lesions whose number was divided in ≤6, 6–20 and >20 as performed in a sub-analysis of the ALSYMPCA study.

Images Acquisition Protocol

Bone scan was acquired 3 h after the injection of 740 MBq of 99mTc-methilen diphosphonate (MDP). The planar whole-body acquisitions were performed by means of a double-head gamma camera (Millennium; GE Healthcare, Milwaukee, WI, USA) that was equipped with a parallel-hole, low-energy, high-resolution collimator on a 10% energy window centered over the 140-keV 99mTc photopeak. Further planar or Single Photon Emission Computed Tomography (SPECT) acquisitions were performed when clinically needed.

18F-Fluorodeoxyglucose Positron Emission Tomography (FDG-PET) was performed according to the international guidelines (1) using a 16-slices Positron Emission Tomography/Computed Tomography (PET/CT) hybrid system (Biograph 16, Siemens Medical Solutions, Knoxville TN, USA). Briefly, the patients fasted overnight prior to the intravenous administration of 300-400 MBq of FDG, which was performed in a quiet room, with the patient lying in a recumbent position and instructed not to move. Blood glucose was measured before tracer injection, so as to ensure blood glucose levels <160 mg/dl. The patients were asked to drink 500 mL of water 1h prior to image acquisition and empty the bladder just before the acquisition start to minimize artifacts caused by the urinary tract. Imaging started 60±15 minutes after intravenous tracer administration. The technical parameters of the 16-detector row, helical CT scanner included a gantry rotation speed of 0.5 s and a table speed of 24 mm per gantry rotation. The PET component of the combined imaging system had an axial view of 16.2 cm per bed position, with an interslice spacing of 3.75 mm. The trans-axial field of view and pixel size of the reconstructed PET images were 58.5 cm and 4.57 mm, respectively, with a matrix size of 128×128. Unenhanced low-dose CT was performed at 140 kV and 40 mA for attenuation correction of emissive data and anatomical localization of the PET dataset. An emissive scan was performed in three-dimensional (3D) mode, shortly after CT acquisition, with a 3-min. acquisition per bed position. PET sinograms were reconstructed by means of ordered-subset expectation maximization (OSEM) iterative reconstruction algorithm (three iterations, eight subsets).

Definition of group response

The FDG-PET data were interpreted according to EORTC and PERCIST criteria by physicians experienced in PET-based response evaluation (MB, SM) that were blinded to ceCT and bone scan results. Response evaluation criteria are detailed elsewhere (2-4) and are briefly summarized below.

Definitions of response group according to EORTC: complete metabolic response (CMR, no FDG uptake within the tumour volume), partial metabolic response (PMR, SUVmax reduction greater than 25% after treatment), stable metabolic disease (SMD, SUVmax increase or decrease less than 25%), progressive metabolic disease (PMD, SUVmax increase greater than 25% or increase in the extension of tumour uptake greater than 20% in the longest dimension or the appearance of new 18F-FDG uptake).

Definition of group response according to PERCIST: FDG-PET data were interpreted according to PERCIST criteria by physicians experienced in PERCIST-based response evaluation. PET readers were blinded to the CT results. PERCIST recommends the use of lean body mass for SUV normalization (SUL). The background area was drawn as a 3-cm-diameter spheric ROI in the right lobe of the liver, as defined in the criteria. The SULpeak of up to five lesions on the baseline and follow-up scan was summed (maximum of two per organ). Target lesions on follow-up scans were not necessarily the same as target lesions at baseline since the hottest lesions were selected in each scan. The five lesions with highest SUL were identified, and a 1.2-cm-diameter spheric ROI was drawn in the hottest part of that lesions. The ROIs were placed in the area of the tumor, where it resulted in the highest possible mean SUL (SULmean). The SULmean of this ROI was defined as SULpeak. The baseline target lesions had to meet the PERCIST 1.0 definition of measurable lesions. The investigators checked that no other lesion could give a higher SULpeak. On subsequent scans, SULpeak could be located in a different lesion from the one measured at baseline, as long as the lesion had been present since baseline. In the follow-up PET/CT scans if SULpeak was decreasing, the response was calculated as ΔSULpeak between baseline and actual follow-up divided by baseline SULpeak X 100%. If SULpeak increased, response was calculated as ΔSULpeak between lowest registered and actual follow-up divided by lowest registered SULpeak x 100%. The response was classified on each scan according to the four categories defined in the criteria set. CMR was complete resolution of 18F-FDG uptake within all lesions to a level less than or equal to that of mean liver activity and indistinguishable from background blood-pool

levels. PMR was a reduction of at least 30% in SULpeak and an absolute drop of 0.8 SULpeak units. PMD was an increase of at least 30% in SULpeak or a new 18F-FDG–avid lesion. SMD was between PMR and PMD.

References

1. Eisenhauer, E.A.; Therasse, P.; Bogaerts, J.; Schwartz, L.H.; Sargent, D.; Ford, R.; Dancey, J.; Arbuck, S.; Gwyther, S.; Mooney, M.; Rubinstein, L.; Shankar, L.; Dodd, L.; Kaplan, R.; Lacombe, D.; Verweij, J. New response evaluation criteria in solid tumours: Revised RECIST guideline (version 1.1). *Eur. J. Cancer* **2009**, *45*, 228–247.
2. Young, H.; Baum, R.; Cremerius, U.; Herholz, K.; Hoekstra, O.; Lammertsma, A.A.; Pruim, J.; Price, P. Measurement of clinical and subclinical tumour response using [18F]-fluorodeoxyglucose and positron emission tomography: review and 1999 EORTC recommendations. *Eur. J. Cancer* **1999**, *35*, 1773–1782.
3. Wahl, R.L.; Jacene, H.; Kasamon, Y.; Lodge, M.A. From RECIST to PERCIST: Evolving Considerations for PET response criteria in solid tumors. *J. Nucl. Med.* **2009**, *50*, 122–150.
4. O, J.H.; Lodge, M.A.; Wahl, R.L. Practical PERCIST: A Simplified Guide to PET Response Criteria in Solid Tumors 1.0. *Radiology* **2016**, *280*, 576–584.
